# The Relative Contributions of Occupational and Community Risk Factors for COVID-19 among Hospital Workers: The HOP-COVID Cohort Study

**DOI:** 10.3390/jcm12031208

**Published:** 2023-02-02

**Authors:** Sylvie Bastuji-Garin, Ludivine Brouard, Irma Bourgeon-Ghittori, Sonia Zebachi, Emmanuelle Boutin, Francois Hemery, Frédéric Fourreau, Nadia Oubaya, Quentin De Roux, Nicolas Mongardon, Slim Fourati, Jean-Winoc Decousser

**Affiliations:** 1 IMRB, INSERM, University Paris Est Creteil, 94010 Créteil, France; 2 Department of Public Health, Hôpitaux Universitaires Henri-Mondor AP-HP, 94010 Créteil, France; 3 Clinical Research Unit (URC Mondor), Hôpitaux Universitaires Henri-Mondor AP-HP, 94010 Créteil, France; 4 CARMAS, University Paris Est Creteil, 94010 Créteil, France; 5 DMU SAPHIRE, Hôpitaux Universitaires Henri-Mondor AP-HP, 94010 Créteil, France; 6 Département d’Information Médicale, Hôpitaux Universitaires Henri-Mondor AP-HP, 94010 Créteil, France; 7 Equipe Opérationnelle d’Hygiène, Département Prévention, Diagnostic, Traitement des Infections, Hôpitaux Universitaires Henri-Mondor AP-HP, 94010 Créteil, France; 8 Service D’Anesthésie-Réanimation Chirurgicale, DMU CARE, Hôpitaux Universitaires Henri-Mondor AP-HP, 94010 Créteil, France; 9 IMRB, EnvA, 94700 Maisons-Alfort, France; 10 Département de Virologie, Bactériologie, Parasitologie-Mycologie, Hôpitaux Universitaires Henri-Mondor AP-HP, 94010 Créteil, France; 11 DYNAMYC, University Paris Est Creteil, 94010 Créteil, France; 12 DYNAMYC, EnvA, 94700 Maisons-Alfort, France

**Keywords:** COVID-19, hospital worker, prevalence, occupational risk factor, geriatric medicine, cluster, community risk factor

## Abstract

The relative contributions of occupational and community sources of COVID-19 among health-care workers (HCWs) are still subject to debate. In a cohort study at a 2814-bed tertiary medical center (five hospitals) in the Paris area of France, we assessed the proportion of hospital-acquired cases among staff and identified risk factors. Between May 2020 and June 2021, HCWs were invited to complete a questionnaire on their COVID-19 risk factors. RT-PCR and serology test results were retrieved from the virology department. Mixed-effects logistic regression was used to account for clustering by hospital. The prevalence of COVID-19 was 15.6% (n = 213/1369 respondents) overall, 29.7% in the geriatric hospitals, and 56.8% of the infections were hospital-acquired. On multivariable analyses adjusted for COVID-19 incidence and contact in the community, a significantly higher risk was identified for staff providing patient care (especially nursing assistants), staff from radiology/functional assessment units and stretcher services, and staff working on wards with COVID-19 clusters among patients or HCWs. The likelihood of infection was greater in geriatric wards than in intensive care units. The presence of significant occupational risk factors after adjustment for community exposure is suggestive of a high in-hospital risk and emphasizes the need for stronger preventive measures—especially in geriatric settings. Clinicaltrials.gov NCT04386759.

## 1. Introduction

Since early 2020, the coronavirus disease 2019 (COVID-19) pandemic has disrupted societies and health-care systems worldwide. As was the case during the epidemic of severe acute respiratory syndrome coronavirus (SARS-CoV-1) infections, health-care workers (HCWs) have paid a high price for their exposure to the pathogen: relative to non-HCWs, the adjusted attack rate ratios for SARS-CoV-2 infection and hospital admission in European countries were 3.0 and 1.8, respectively [1]. These elevated ratios might have been due to dual exposure (i.e., occupational and community) and probably resulted in disease transmission to both patients and fellow workers [2]. However, the literature data on the relative contributions of occupational and community sources of infection are contradictory. Whereas several studies (including some with SARS-CoV-2 genotyping data) reported that most infections were community-acquired, others stressed the importance of occupational risk factors [2,3,4,5,6,7,8,9,10,11,12,13,14,15,16,17,18]. Furthermore, very few studies have focused on staff in geriatric wards and nursing homes [19,20], even though outbreaks have been reported in these settings [21,22,23].

It was recently suggested that gaining better knowledge of occupational infection and associated risk factors is critically important for the development and implementation of preventive measures in high-risk HCW populations [24]. This issue is especially relevant when human resources and material resources (e.g., personal protective equipment (PPE)) are scarce and must be optimized.

In the present cohort study, we collected data on the personal and occupational characteristics of hospital staff working in different settings in the same hospital group during the first three waves of the COVID-19 pandemic in France. The study’s objectives were to determine the relative weights of occupational and nonoccupational sources of infection and to identify occupational risk factors.

## 2. Material and Methods

### 2.1. Study Design and Participants

Between 18 May 2020 and 16 June 2021, we conducted a cohort study with prospective recruitment at Henri Mondor University Medical Center, a 2814-bed tertiary care center belonging to the Greater Paris University Hospitals (Assistance Publique des Hôpitaux de Paris). This referral center has five hospitals (of which three are dedicated to geriatric medicine) and provides acute care, rehabilitation, and long-term care. During the first recruitment period, all HCWs (caregivers and non-caregivers) were invited by email or via posters to participate in the survey. The moderate recruitment rate and the greater availability of real-time reverse-transcriptase polymerase chain reaction (RT-PCR) tests prompted us to modify the study protocol. Hence, in a second recruitment period (from 5 November 2020), we sought to recruit only HCWs with RT-PCR test data. Given that some HCWs had been vaccinated by February 2021, we split the second recruitment period into two parts (before and after 1 March 2021). Our study was initially designed to (i) retrospectively collect information for the first epidemic wave (starting in March 2020), and (ii) prospectively study weekly self-questionnaire data from HCWs during the subsequent pandemic period.

Prior to inclusion, all participants gave informed consent for use of their personal data. The study was approved by an institutional review board (CPP Ile-de-France IV, Paris, France, 2020/45) and was registered at Clinicaltrials.gov (NCT04386759).

### 2.2. Data Collection and Study Variables

Although a hospital-wide, active monitoring program had not been implemented, HCWs were asked to complete a web-based, standardized, electronic case report form (CleanWEB^®^, Telemedicine Technologies SAS, Boulogne-Billancourt, France) regarding their COVID-19 status and risk factors. The questions concerned the occurrence (from March 2020 onwards) of COVID-19 symptoms (fever, cough, anosmia, ageusia, and other suggestive symptoms), a clinical diagnosis of COVID-19 with sick leave, and the dates and results of RT-PCR tests. Given that all the staff were invited to undergo a SARS-CoV-2 serology test in May 2020, the latter results and the RT-PCR results were retrieved from the center’s virology lab.

Potential risk factors collected included demographic variables, characteristics of the domestic environment, work activities, and characteristics of the work environment. These data were collected for the two weeks prior to the COVID-19 symptom onset, a diagnosis of COVID-19, an RT-PCR test, or inclusion in the study. Depending on their work activities and workplace, the hospital staff were divided into four categories: (1) patient care (e.g., physicians, nurses and allied health professionals working in clinical wards), (2) patient contact without care provision (e.g., nurse managers, receptionists, and hospital porters), (3) contact with caregivers (e.g., cleaning staff, nursery staff, and some administrative staff), and (4) little or no contact with caregivers (e.g., researchers, catering staff, and administrative managers). For logistic regression modeling, medical departments with limited patient contact constituted the reference category for the “workplace” variable. We hypothesized that some administrative or technical staff were nevertheless at risk of infection, and so the group with the lowest COVID-19 prevalence (≤10%) constituted the reference category for the job category variable.

For each department, we also retrospectively collected monthly administrative data on the presence of COVID-19 clusters among the patients and/or the staff and the number and proportion of COVID-19 patients. The latter data were available for inpatient wards other than psychiatry, rehabilitation, and long-term care. Lastly, daily data on the incidence of COVID-19 in the counties where the HCWs lived were collected from the French government’s databases (https://www.data.gouv.fr/fr/datasets/indicateurs-de-suivi-de-lepidemie-de-covid-19/, accessed on 1 November 2021). These data became available on 19 May 2020. The daily incidence was then averaged over a month, expressed per 100,000 inhabitants, and categorized as follows: <50, 50 to 150, 150 to 250, ≥250, or not available.

### 2.3. Outcome

The study outcome was the participants’ COVID-19 status. At the end of the study, each participant was classified as (i) not having had COVID-19, (ii) having had a definite diagnosis of COVID-19, or (iii) having had a probable diagnosis of COVID-19. A definite diagnosis of COVID-19 was defined as a positive RT-PCR or serology test. A probable diagnosis of COVID-19 was defined as the presence of suggestive symptoms, sick leave for COVID-19, and the absence of both RT-PCR and serology test results, or test results that were not relevant. Indeed, a negative RT-PCR test result 2 or more days before symptom onset or 10 days or more days afterwards or a negative serology test result less than 10 days after symptom onset were not considered to be relevant. All other participants were considered not to have been infected.

We also assessed whether the infection had probably been acquired at the hospital or elsewhere. Community-acquired infection was considered to be very likely in people who were teleworking only or who had been in contact with COVID-19 cases outside work only. Hospital-acquired infection was considered to be very likely in people who had been in contact with COVID-19 cases at work only. Other infections were considered to be of undetermined origin.

### 2.4. Statistical Analysis

Continuous variables with a skewed distribution were log-transformed. The corresponding odds ratios (ORs) [95% confidence interval (CI)] were quoted for a standard deviation increment in the log-transformed value.

The frequency of a definite or probable diagnosis of COVID-19 was estimated, together with its 95% CI. We compared participants with vs. without COVID-19 using a Mann–Whitney, chi-squared, or Fisher test, as appropriate. The crude ORs [95% CI] were estimated in asymptotic logistic regression analyses for variables with *p* < 0.15. Next, multivariable ORs were estimated using mixed-effects logistic regression modeling with a random intercept (to account for correlation within each hospital). To avoid the introduction of strongly correlated variables into the multivariable models, we assessed correlations using Cramer’s V. Multivariable models were systematically adjusted for the recruitment period, the regional incidence of COVID-19, and COVID-19 contact in the community. Lastly, two sensitivity analyses were carried out by (i) using multiple imputations of missing data, and (ii) after excluding staff with a probable diagnosis of COVID-19. After checking compliance with the missing-at-random hypothesis (by exploring the missingness pattern), we used the multiple-multivariate imputations-by-chained-equations procedure in STATA software (version 15.0, StataCorp, College Station, TX, USA) to estimate missing values for covariates associated with COVID-19 status.

The hypothesis testing was two-sided, and results were considered to be statistically significant when the 95% CI did not encompass the value of 1. Analyses were performed with STATA software.

## 3. Results

### 3.1. Participants

**Figure 1:**
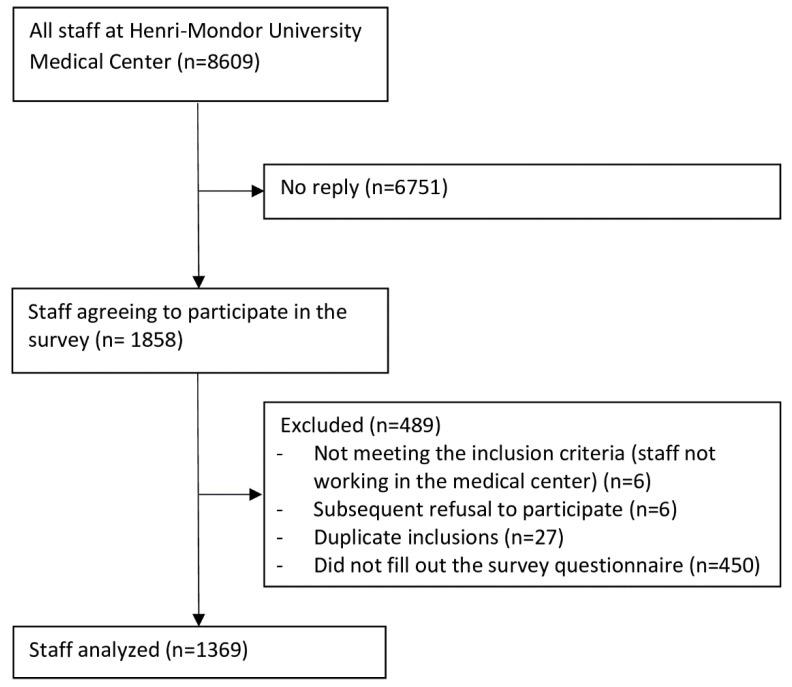
Flowchart of the HOP-COVID survey.

Among 8609 HCWs, 1858 agreed to participate and 1369 (15.9% of the total) filled out the questionnaire (Figure 1). In sum, 263 respondents (19.2%) worked in one of the three geriatric hospitals. The median (range) age was 43 (19–71) years, and 72.6% of the participants were women. Physicians, nurses, nursing assistants, and allied health professionals (representing about 80% of the HCWs in our hospital) accounted for 62.1% (n = 849) of the participants. When considering staff in clinical wards (n = 809), 26% worked in geriatric wards, 16.4% in intensive care units (ICU), 11.4% in emergency departments, 8.8% worked in surgical wards, and 37.5% in other wards.

### 3.2. Outcome

During the study period, 770 (56.3%) staff had undergone an RT-PCR test and 893 (65.2%) had undergone a serology test. The test results enabled us to classify 1055 (77.1%) participants. Of these, 213 (15.6%; [95%CI]: 13.6–17.5%) had a definite diagnosis (n = 175) or a probable diagnosis (n = 38) of COVID-19 (Table 1). COVID-19 was probably hospital-acquired in 121 (56.8%) cases.

Staff working in a geriatric hospital were more likely than other staff to have had COVID-19 (29.7% vs. 12.2%, respectively; OR [95% CI]: 3.0 [2.2–4.2]). On univariable analysis (Table 2), the proportion of staff with COVID-19 was significantly higher when the regional daily incidence of COVID-19 was ≥50/100,000 inhabitants. There were no significant differences between the classes. Accordingly, incidence classes were dichotomized as <50/100,000 vs. >50/100,000 (OR [95% CI]: 7.5 [4.2–13.3]). COVID-19 contact in the community was associated with a higher likelihood of infection. When considering occupational factors (Table 3), we found that non-teleworking HCWs had a significantly higher risk of infection. We also observed significant differences between occupations: physicians, nurses, nursing assistants, and some administrative staff (emergency call handlers/dispatchers, receptionists, nursery staff, and secretaries) had higher infection rates. The highest proportions of COVID-19 were observed for staff in geriatric wards, radiology/functional assessment facilities, and stretcher services. There were no significance differences between the types of geriatric ward (acute, rehabilitation, or long-term care). Staff providing patient care and those working in wards with COVID-19 patients or COVID-19 clusters were more likely to have had COVID-19. In clinical wards, this risk increased significantly with the number and proportion of COVID patients.

**Figure 2:**
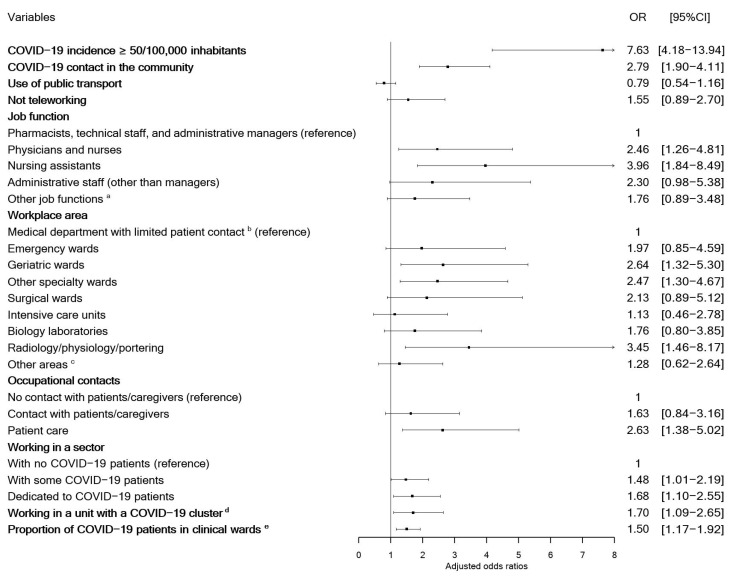
Estimated adjusted odds ratios by recruitment period, regional incidence of COVID-19, COVID-19 contact in the community, and correlations within each hospital. Adjusted odds ratios were estimated using mixed-effects logistic regression modeling with a random intercept (to account for correlation within each hospital); the intraclass correlation coefficient was 0.09 (standard deviation: 0.21). Dots indicate mean estimates, horizontal lines, 95% CI. ^a^ Other job functions include nurse managers, allied health professionals, students, laboratory technicians, administrative staff in clinical units, researchers, and research support staff; ^b^ medical departments with limited patient contact include hospital pharmacies, hemovigilance, pharmacovigilance center, infection control units, IT departments, public health department, research support units, and research center; ^c^ other areas include occupational health services, teleconsultation facilities, nursing schools, nurseries, administrative units, and technical services. ^d^ The proportion of COVID-19 patients was determined for all inpatient wards other than psychiatry, rehabilitation and long-term care (N = 579); ^e^ ORs (95% CI) were quoted for one standard deviation increment in the log-transformed value.

Adjustment for the recruitment period, regional COVID-19 incidence, COVID-19 contacts in the community, and within-hospital correlations did not modify the nature of the associations between COVID-19 on one hand and job function (physicians, nurses, nursing assistants, and administrative staff), workplace area (geriatric wards, other specialist wards, radiology/functional assessment facilities, and stretcher services), providing patient care, and working in a sector with COVID-19 patients or clusters or with a high proportion of COVID-19 patients on the other (Figure 2). It is noteworthy that the regional incidence was the greatest risk factor. The sensitivity analyses (after multiple imputations or after exclusion of HCWs with a probable diagnosis) gave similar results (Table S1). After additional adjustments for working in a clinical ward with COVID-19 patients or for the proportion of COVID-19 patients, working on a geriatric ward or another specialist ward remained significantly associated with infection relative to ICUs (Table S2). The strong correlations between the variables (specifically job functions, workplace areas, and patient contacts, all of which gave Cramer’s V ≥ 0.49) prevented us from building a multivariable model accounting for all the variables.

## 4. Discussion

We found a high prevalence of COVID-19 among HCWs in general (15.6%) and among staff working in geriatric settings in particular (29.7%). More than half of the cases of COVID-19 were probably hospital-acquired. After adjustment for the regional incidence of COVID-19 and COVID-19 contacts in the community, care providers (especially nursing assistants) and staff in units with COVID-19 patients or clusters had a greater risk of COVID-19 than other HCWs. This was also true for non-caregivers in radiology/functional assessment facilities and stretcher services. The likelihood of infection was greater in specialist wards than in ICUs.

The prevalence of COVID-19 among hospital staff observed here (15.6%) was higher than that reported in the literature. A meta-analysis of cases of COVID-19 during the first wave estimated that 8.5% of European HCWs were seropositive for SARS-CoV-2 [25]. However, a recent study that included HCWs from November 2020 to February 2021 reported a 13% seroprevalence among unvaccinated individuals [16]. Thus, our unexpectedly high prevalence might be due to our inclusion period during the first three waves of COVID-19 in France (increasing the cumulative incidence) and by the value of 29.7% observed in geriatric hospitals. Values of 45.8% and 62.6% were reported in the only two studies of geriatric settings [19,20]. However, given the moderate participation rate, our prevalence estimates—and especially those for the geriatric hospitals—should be interpreted with caution.

More than half of the cases of COVID-19 (56.8%) were probably hospital-acquired. This rate is higher than those previously reported in the literature (ranging from 25% to 48%) on the basis of self-reported infections [9,10,14,26]. Several studies found that exposure in a community setting was the greatest risk factor [27], and recent studies with genotyping data have shown that the community is the main source of SARS-CoV2 infection [3,4,5]. However, these molecular approaches are often incomplete and tend to focus on a small number of infections. Given the absence of virological tracing of all the contacts in the present study (as in most literature studies), our assessment of the source of contamination is subject to a degree of uncertainty. However, our detection of several objectively reported occupational risk factors (such as working in a ward dedicated to COVID-19 patients or in a sector with COVID-19 clusters) after adjustment for regional incidence and community exposure suggests that the in-hospital risk was high. Unfortunately, we were not able to differentiate between HCW-to-HCW transmission and patient-to-HCW transmission.

In line with previous reports of a significant relationship between COVID-19 incidence and the occurrence of COVID-19 cases among HCWs [6,28], we observed an eightfold greater risk of infection when the regional incidence exceeded 50/100,000 inhabitants. High incidence of COVID-19 not only promotes infections through community contacts but also increases the number of hospitalized cases and thus the risk of in-hospital infection.

As reported previously, frontline HCWs (particular the nursing assistants, in our study) were more likely to develop COVID-19 [7,8,13,15,16,25,28,29,30]. Moreover, the elevated risks associated with working in dedicated COVID-19 wards and with a high proportion of COVID-19 patients (independently of the type of ward) are consistent with the previously reported quantitative relationship between exposure to COVID-19 patients and the likelihood of a positive RT-PCR test [29]. Therefore, wards should probably commit to more effective use of PPE. Despite the higher proportion of COVID-19 patients and the higher frequency of high-risk medical procedures in ICUs, the staff in these units were not at greater risk of COVID-19 than staff in other wards. This finding is in line with several previous reports [7,28,31]. The absence of greater risk in the ICU is probably due to greater vigilance, the systematic use of masks, and the use of long-standing, standardized PPE procedures. Less experienced units might not be as vigilant or might not have similar procedures in place. Furthermore, combating in-hospital infections requires active commitment by the patient (e.g., by wearing a mask and by complying with self-isolation instructions). Many geriatric patients are doubtless unable or unwilling to comply with protective measures and so might infect HCWs more readily. This issue has been emphasized during outbreaks in geriatric wards [21]. In our study, the likelihood of COVID-19 was significantly higher in geriatric hospitals than in other hospitals. Even after taking into account the hospital site, the risk of COVID-19 was four times higher in geriatric wards than in the ICUs. The only two published studies of geriatric hospitals also reported high infection rates (45.8% and 62.6%) [19,20]. Hence, one of our study’s most important messages is that protective measures should be focused on HCWs working in geriatric hospitals and geriatric units.

In line with a few previous studies, we found that non-caregivers in contact with patients and/or caregivers (notably staff in radiology, functional assessment and stretcher services) had a higher risk of COVID-19 than other staff [14,32,33]. Indeed, staff can be exposed to SARS-CoV-2 before the infected patient has received a firm diagnosis. Furthermore, non-caregivers might be less knowledgeable about PPE use. During the first wave (before RT-PCR testing became widely available), a CT scan was a first-line option for diagnosing COVID-19 [34].

In line with a recent report, we found that HCWs working in wards with COVID-19 clusters had a greater likelihood of infection. This reflects uncontrolled in-hospital transmission of the virus [28]. We can therefore assume that the infection control measures implemented in our hospital network were not stringent enough. Not all wards housing COVID-19 patients (especially geriatric wards) were fitted with negative air pressure systems. Although supplies of masks, FFP2 respirator masks, gloves, gowns, and goggles were tight, no shortages occurred. During the study period, each HCW had at least two new surgical masks per day. However, in line with national guidelines, the medical center did not supply FFP2 respirator masks to all wards housing COVID-19 patients, but focused on high-risk situations (e.g., aerosol-generating procedures).

## 5. Limitations

Our study had several limitations. Firstly, the modest participation rate means that recruitment bias cannot be ruled out. The lower perceived risk among nonclinical staff might have led to a lower participation rate and thus an overestimation of the in-hospital risk. However, the inclusion of remote workers and the slight underrepresentation of physicians, nurses, nursing assistants, and allied health professionals in our study (accounting for 62.1% of the study participants but 80% of all hospital staff) argue against this hypothesis. Secondly, given that vaccination started in late January 2021 in our hospital, it is possible that fully vaccinated staff (i.e., having received two doses and thus protected from March 2021 onwards) were included in the study. Unfortunately, this information was not available in the CRFs. However, considering that (i) only a small proportion (6.5%) of the study population was included between March and July 2021 and (ii) all the analyses were adjusted for this recruitment period, we do not believe that vaccination significantly altered our results. Thirdly, one in six of the participants with COVID-19 had a probable (but not definite) diagnosis. However, any misclassification of positive diagnosis should have been the same for all work sectors and categories. Further, the results of our sensitivity analyses supported our main findings. Fourthly, we did not assess the workers’ level of knowledge or compliance with infection control measures. Given that exposure was self-reported, we cannot therefore rule out the possibility of recall bias. Lastly, the limited number of cases and the interdependence of the variables analyzed prevented us from analyzing all the possible risk factors in a single model. Hence, our findings should be confirmed in larger studies with more details at the ward level (and especially in geriatric wards). Nevertheless, one must be aware that the interpretation of future studies will be blurred by (i) the spread of SARS-CoV-2 variants with differences in virulence and transmissibility, and (ii) the impact of COVID-19 preventive strategies.

## 6. Conclusions

The presence of significant occupational risk factors after adjustment for community risk factors suggests that the in-hospital risk is elevated. Our results emphasize that preventive measures (training and the allocation of material and human resources) must be focused on the most at-risk HCWs, especially in geriatric settings. In the context of an emerging, highly contagious disease, caring for older patients with COVID-19 is a challenge for HCWs.

## Figures and Tables

**Table 1:** General characteristics of the 213 hospital workers with a definite or a probable diagnosis of COVID-19.

	Total	Diagnosis of COVID-19		
		Definite	Probable	*p*-Values ^a^
	N = 213	N = 175	N = 38	
Fever and respiratory symptoms	93 (43.9)	76 (43.7)	17 (44.7)	0.91
Anosmia and/or ageusia	89 (42.0)	76 (43.7)	13 (34.2)	0.28
Other symptoms ^b^	159 (74.6)	127 (72.6)	32 (84.2)	0.15
SARS-CoV-2 RT-PCR test				
Not performed	49 (23.0)	30 (17.1)	19 (50.0)	-
Not helpful ^c^	26 (12.2)	7 (4.0)	19 (50.0)	
Negative ^d^	3 (1.4)	3 (1.7)	0 (-)	
Positive	135 (63.4)	135 (77.1)	0 (-)	
SARS-CoV-2 serology test				
Not performed	84 (39.4)	52 (29.7)	32 (84.2)	-
Not helpful ^e^	58 (27.2)	52 (29.7)	6 (15.8)	
Negative	6 (2.8)	6 (3.4)	0 (-)	
Positive	65 (30.5)	65 (37.1)	0 (-)	
Patient classification				
Positive RT-PCR test	110 (51.6)	110 (62.9)	0 (-)	-
Positive RT-PCR and serology tests	25 (11.7)	25 (14.3)	0 (-)	
Positive serology test	40 (18.8)	40 (22.9)	0 (-)	
Clinical diagnosis/sick leave	38 (17.8	0 (-)	38 (100)	
COVID-19 acquisition				0.59
Probably hospital-acquired	121 (56.8)	99 (56.6)	22 (57.9)	
Probably community-acquired	51 (23.9)	44 (25.1)	7 (18.4)	
Indefinite ^f^	41 (19.3)	32 (18.3)	9 (23.7)	

Abbreviations: RT-PCR, real-time reverse-transcriptase polymerase chain reaction. The data are quoted as the number (col %). ^a^ *p* value for a chi-squared test. ^b^ Other symptoms include myalgia, arthralgia, headache, diarrhea, or unusual fatigue. ^c^ A negative RT-PCR test was not considered to be of diagnostic value if it was performed more than 2 days before symptom onset or more than 10 days after. ^d^ All three had a positive serology test. ^e^ Only negative serology tests performed at least 10 days after symptom onset or inclusion date were considered to be of diagnostic value. ^f^ Indefinite acquisition corresponded to the 7 hospital workers who mentioned both occupational and community sources of infection and the 34 for whom no specific source of infection was identified (although the workers did confirm that they were not teleworking).

**Table 2:** General characteristics and community exposure of the 1369 hospital workers included in the study as a function of their COVID-19 status (univariable analysis).

		Diagnosis of COVID-19			
	Total	No	Definite/Probable	*p*	
Characteristics	(n = 1369) ^a^	(n = 1156) ^b^	(n = 213) ^b^	Value ^c^	OR [95% CI]
**Recruitment period**				0.003	
18 May 2020–5 November 2020	809 (59.1)	700 (86.5)	109 (13.5)		1.4 [0.7–2.8]
6 November 2020–28 February 2021	471 (34.4)	376 (79.8)	95 (20.2)		2.3 [1.1–4.6]
1 March 2021–16 July 2021	89 (6.5)	80 (89.9)	9 (10.1)		Ref.
**Hospital** (n = 1368, 1155/213)				<0.001	
Henri Mondor	1000 (73.1)	877 (87.7)	123 (12.2)		0.8 [0.3–1.7]
Albert Chenevier	105 (7.7)	93 (88.6)	12 (11.4)		Ref.
Emile Roux (geriatric hospital)	123 (9.0)	83 (67.5)	40 (32.5)		3.4 [2.3–5.2]
Joffre-Dupuytren (geriatric hospital)	68 (5.0)	43 (63.2)	25 (36.8)		4.2 [2.5–7.0]
Georges Clemenceau (geriatric hospital)	72 (5.3)	59 (81.9)	13 (18.1)		1.4 [0.5–3.6]
**Regional daily COVID-19 incidence per 100,000 inhabitants**				<0.001	
<50	392 (28.6)	379 (96.7)	13 (3.3)		Ref.
50 to 149	157 (11.5)	130 (82.8)	27 (17.2)		6.1 [3.0–12.1]
150 to 249	140 (10.2)	120 (85.7)	20 (14.3)		4.9 [2.4–10.1]
≥250	246 (18.0)	194 (78.9)	52 (21.1)		7.8 [4.1–14.6]
Before data were available (May 2020)	434 (31.7)	333 (76.7)	101 (23.3)		8.8 [4.9–16.1]
**Age, median [IQR], years** (n = 1368, 1155/213)	43 [32–53]	43 [32–53]	43 [33–53]	0.68	-
**Sex**				0.46	
Male	375 (27.4)	321 (85.6)	54 (14.4)		
Female	994 (72.6)	835 (84.0)	159 (16.0)		-
**Blood group** (n = 1221, 1036/185)				0.31	
O	517 (42.3)	445 (86.1)	72 (13.9)		-
A, B or AB	704 (57.7)	591 (83.9)	113 (16.0)		
**Living with children attending primary school** (n = 1361, 1149/212)				0.22	
No	971 (71.4)	827 (85.2)	144 (14.8)		-
Yes	389 (28.6)	321 (82.6)	68 (17.5)		
**Living with children attending junior high or high school** (n = 1361, 1149/212)				0.53	
No	964 (70.9)	810 (84.0)	154 (16.0)	.	-
Yes	396 (29.1)	338 (85.4)	58 (14.6)		
**Use of public transport** (n = 1368, 1155/213)				0.15	
No	1010 (73.9)	844 (83.6)	166 (16.4)		Ref.
Yes	357 (26.1)	310 (86.8)	47 (13.2)		0.8 [0.5–1.1]
**COVID-19 contact in the community** (n = 1352,1139/213)				<0.001	
No	1167 (86.3)	1012 (86.7)	155 (13.28)		Ref.
Yes	185 (13.7)	127 (68.65)	58 (31.35)		3.0 [2.1–4.3]

Abbreviations: OR, odds ratio; CI, confidence interval; IQR, interquartile range. The data are quoted as the number (%), unless otherwise stated. The format (n = X, Y/Z) indicates the total number of staff with data (X), the number of non-COVID-19 staff with data (Y), and the number of COVID-19 staff with data (Z). ^a^ Percentages correspond to the percentage of the column; ^b^ percentages correspond to the percentage of the row. ^c^ *p* values for a chi-squared, Fisher’s exact, or Mann–Whitney tests, as appropriate; the ORs [95% CI] were estimated in a univariable logistic regression.

**Table 3:** The occupational environment of the 1369 hospital workers included in the study as a function of their COVID-19 status (univariable analysis).

		Diagnosis of COVID-19			
	Total	No	Definite/Probable	*p*	
Characteristics	(n = 1369) ^a^	(n=1156) ^b^	(n = 213) ^b^	Value ^c^	OR [95% CI]
**Activity** (n = 1359, 1146/213)				0.06	
Teleworking	49 (3.6)	45 (91.8)	4 (8.2)		Ref.
Alternate teleworking and in-hospital work	127 (9.4)	114 (89.8)	13 (10.2)		
In-hospital work	1183 (87.0)	987 (83.4)	196 (16.6)		1.9 [1.1–3.1]
**Job function** (n = 1367, 1154/213)				0.003	
Physicians	374 (27.4)	311 (83.2)	63 (16.8)		2.2 [1.2–4.1]
Nurses	157 (11.5)	131 (83.4)	26 (16.8)		
Nursing assistants	115 (8.4)	83 (72.2)	32 (27.8)		4.1 [2.0–8.5]
Nurse managers	102 (7.5)	90 (88.2)	12 (11.8)		1.6 [0.9–3.1]
Allied health professionals ^d^	101 (7.4)	88 (87.1)	13 (12.9)		
Medical and other students	94 (6.9)	83 (88.3)	11 (11.7)		
Laboratory technicians	57 (4.2)	49 (86.0)	8 (14.0)		
Administrative staff in clinical units	73 (5.3)	62 (84.9)	11 (15.1)		
Researchers and research support staff	63 (4.6)	54 (85.7)	9 (14.3)		
Pharmacists	30 (2.2)	28 (93.3)	2 (6.7)		Ref.
Technical and administrative unit managers	61 (4.5)	56 (91.8)	5 (8.2)		
Technical staff (other than managers)	50 (3.7)	45 (90.0)	5 (10.0)		
Administrative staff (other than managers)	90 (6.6)	74 (82.2)	16 (17.8)		2.3 [1.0–5.2]
Workplace area (n = 1319, 1106/213)				0.02	
Emergency departments	92 (7.0)	80 (87.0)	12 (13.0)		1.3 [0.6–2.9]
Geriatric wards	210 (15.9)	150 (71.4)	60 (28.6)		3.5 [1.9–6.4]
Other specialty wards	303 (23.0)	250 (82.5)	53 (17.5)		1.9 [1.1–3.4]
Surgical wards	71 (5.4)	60 (84.5)	11 (15.5)		1.6 [0.7–3.7]
Intensive care units	133 (10.1)	124 (93.2)	9 (6.8)		0.6 [0.3–1.5]
Medical biology laboratories	125 (9.5)	109 (87.2)	16 (12.8)		1.3 [0.6–2.7]
Radiology/physiology/functional assessment facilities	50 (3.8)	40 (80.0)	10 (20.0)		2.6 [1.1–5.7]
Stretcher services	8 (0.6)	5 (62.5)	3 (37.5)		
Medical departments with limited patient contact ^e^	157 (11.9)	141 (89.8)	16 (10.2)		Ref.
Other services ^f^	170 (12.9)	147 (86.5)	23 (13.5)		1.4 [0.7–2.7]
**Type of geriatric ward** (n = 197, 148/49)				0.90	
Acute care ward	65 (33.0)	50 (76.9)	15 (23.1)		-
Rehabilitation ward	103 (52.3)	76 (73.8)	27 (26.2)		
Long-term care ward	29 (14.7)	22 (75.9)	7 (24.1)		
**Type of laboratory** (n = 124, 109/15)				0.13	
Microbiology	42 (33.9)	35 (83.3)	7 (16.7)		2.4 [0.8–7.4]
Pathology	18 (14.5)	15 (83.3)	3 (16.7)		
Other	64 (51.6)	59 (92.2)	5 (7.8)		Ref
**Contact with patients or caregivers** (n = 1358, 1145/213)				0.006	
Patient care	622 (45.8)	502 (80.7)	120 (19.3)		2.1 [1.2–3.9]
Patient contact without care provision	255 (18.8)	218 (85.5)	37 (14.5)		1.3 [0.7–2.5]
Contact with caregivers	355 (26.1)	310 (87.3)	43 (12.1)		
No or limited contact with caregivers	128 (9.4)	115 (89.8)	13 (10.2)		Ref.
COVID-19 sectors (n = 1319, 1108/201)				0.045	
No COVID-19 patients	782 (59.3)	680 (86.0)	102 (13.0)		Ref.
Some COVID-19 patients	288 (21.8)	236 (81.9)	52 (18.1)		1.5 [1.0–2.1]
Dedicated to COVID-19 patients	249 (18.9)	202 (81.1)	47 (18.9)		1.6 [1.1–2.3]
**COVID-19 patient burden**, median [IQR] (n = 579, 482/97) ^g^					
**Number of COVID-19 patients**	4 [1–15]	4 [1–11]	8 [3–29]	<0.001	1.4 [1.1–1.8]
**Proportion of COVID-19 patients**	6.1 [2–31]	6 [2–27]	19.1 [5–51]	<0.001	1.5 [1.2–1.9]
**Working in a sector with COVID-19 clusters (n = 1164, 1156/213)**				<0.001	
No	981 (71.7)	863 (88.0)	118 (12.0)		Ref.
Cluster among the patients	29 (2.1)	18 (62.1)	11 (37.9)		2.1 [1.4–3.1]
Cluster among the staff	67 (4.9)	55 (82.1)	12 (17.9)		
Cluster among the patients and staff	87 (6.4)	69 (73.9)	18 (20.7)		
**Systematic use of a mask in hospital** (n = 1334, 1127/207)				0.59	
No	143 (10.7)	123 (86.0)	20 (14.0)		-
Yes	1191 (89.3)	1004 (84.3)	187 (15.7)		

Abbreviations: OR, odds ratio; CI, confidence interval; IQR, interquartile range. The data are quoted as the number (%), unless otherwise stated. The format (n = X, Y/Z) indicates the total number of staff with data (X), the number of non-COVID-19 staff with data (Y), and the number of COVID-19 staff with data (Z). ^a^ Percentages correspond to the percentage of the column; ^b^ percentages correspond to the percentage of the row. ^c^ *p* values for chi-squared, Fisher’s exact, or Mann–Whitney tests, as appropriate; the ORs [95% CI] were estimated in a univariable logistic regression. ^d^ Allied health professionals included physiotherapists, occupational therapists, podiatrists, speech therapists, psychologists, dieticians, and social workers. ^e^ Medical departments with limited patient contact were hospital pharmacies, hemovigilance, pharmacovigilance, infection control units, IT departments, public health department, research support units, and research center. ^f^ Other areas include occupational health-care services, teleconsultation facilities, nursing schools, nurseries, and administrative and technical departments. ^g^ The proportion of COVID-19 patients was available for all inpatient wards other than psychiatry, rehabilitation, and long-term care; ORs [95% CI] were quoted for one standard deviation increment in the log-transformed value.

## Data Availability

The data presented in this study are available on request from the corresponding author. The data are not publicly available due to privacy.

## Supplementary Materials

The following supporting information can be downloaded at: https://www.mdpi.com/article/10.3390/jcm12031208/s1, Table S1. Sensitivity analyses of COVID-19 among hospital workers as function of personal and occupational characteristics (mixed-effects logistic regression modeling accounting for correlation within each hospital). Table S2. Results of multivariable analyses of hospital workers from clinical wards (mixed-effects logistic regression modeling, accounting for correlation within each hospital).
