# Supplementary material for: The Relative Contributions of Occupational and Community Risk Factors for COVID-19 among Hospital Workers: The HOP-COVID Cohort Study
Source: J Clin Med. 2023 Feb 2;12(3):1208. doi: 10.3390/jcm12031208 (PMC9917902; doi:10.3390/jcm12031208)
Supplement: Supplementary file 1 [file jcm-12-01208-s001.zip › jcm-2158022-supplementary.pdf]

Table S1. Sensitivity analyses of COVID-19 among hospital workers as function of personal and occupational characteristics (mixed-effects logistic regression modeling accounting for correlation within each hospital)

Characteristics	Definite or probable diagnosis of COVID-19 vs. no COVID-19 after multiple imputations (n=213/1156) OR [95%CI] [a]	Definite diagnosis of COVID-19 vs. no COVID-19 (n=175/1138) OR [95%CI] [a]
**Recruitment period**		
May 18–November 5, 2020	2.7 [1.3–5.8]	2.0 [0.9–4.3]
November 6, 2020–February 28, 2021	2.01 [1.0–4.3]	1.9 [0.9–4.0]
March 1, 2021- July 16, 2021	Ref.	Ref.
**Regional daily COVID-19 incidence per 100,000 inhabitants**		
<50	Ref.	Ref.
≥50	7.7 [4.2–14.0]	10.6 [4.8–23.3]
**COVID-19 contact in the community**		
No	Ref.	Ref.
Yes	2.8 [1.9–4.2]	2.9 [1.9–4.3]
**Use of public transport**		
No	Ref.	Ref.
Yes	0.8 [0.5–1.1]	0.8 [0.5–1.2]
**Teleworking**		
No	1.6 [0.9–2.7]	1.6 [0.8–2.9]
Yes	Ref.	Ref.
**Job function**		
Pharmacists/technical staff/administrative managers	Ref.	Ref.
Physicians and nurses	2.4 [1.3–4.8]	2.4 [1.2–4.9]
Nursing assistants	3.9 [1.8–8.4]	3.5 [1.6–7.9]
Administrative staff (other than managers)	2.3 [1.0–3.5]	2.0 [0.8–5.1]
Other job functions [b]	1.8 [0.9–3.5]	1.5 [0.7–3.1]
**Workplace area**		
Medical department with limited patient contact [c]	Ref.	Ref.
Emergency departments	2.0 [0.9–4.7]	2.16 [0.8–5.7]
Geriatric wards	2.7 [1.4–5.4]	3.30 [1.5–7.3]
Other specialty wards	2.5 [1.3–4.7]	3.29 [1.6–6.8]
Surgical wards	2.1 [0.9–5.1]	2.76 [1.1–7.2]
Intensive care units	1.1 [0.5–2.7]	0.93 [0.3–2.9]
Medical biology laboratories	1.8 [0.8–3.9]	1.50 [0.6–3.8]
Radiology/functional assessment/portering	3.5 [1.5–8.3]	4.4 [1.7–11.5]
Other areas [d]	1.3 [0.7–2.8]	1.4 [0.59–3.2]
**Occupational contacts**		
No contact with patients/caregivers	Ref.	Ref.
Contact with patients/caregivers	1.6 [0.9–3.2]	2.6 [1.1–6.1]
Patient care	2.6 [1.4–5.0]	4.2 [1.8–9.8]
**Working in a sector**		
With no COVID-19 patients	Ref.	Ref.

With some COVID-19 patients	1.5 [1.1–2.2]	1.5 [1.0–2.3]
Dedicated to COVID-19 patients	1.7 [1.1–2.6]	1.9 [1.2–2.9]
**Working in a unit with COVID-19 clusters**		
No	Ref.	Ref.
Yes	1.8 [1.2–2.7]	1.8 [1.1–2.9]
**Proportion of COVID-19 patients in clinical wards[e]**	1.7 [1.3–2.1]	1.5 [1.2–2.0]

Abbreviations: OR, odds ratio; CI, confidence interval. [a] OR for the association between the specified factor and COVID-19 using mixed-effects logistic regression (with a random intercept) after adjustment for recruitment period, regional COVID-19 incidence, COVID-19 contact in the community and accounting for correlation within each hospital. [b] Other job functions include nurse managers, allied health professionals, students, laboratory technicians, administrative staff in clinical units, researchers and research support staff. [c] Medical departments with limited patient contact include hospital pharmacies, hemovigilance, pharmacovigilance center, infection control units, IT departments, public health departments, research support units, and research centers. [d] Other areas include occupational health services, teleconsultation facilities, nursing schools, nurseries, administrative units, and technical services. [e] The proportion of COVID-19 patients was determined for all inpatient wards other than psychiatry, rehabilitation, and long-term care (N=579); ORs [95%CI] are quoted for one standard deviation increment in the log-transformed value.

Table S2. Results of multivariable analyses of hospital workers from clinical wards (mixed-effects logistic regression modeling, accounting for correlation within each hospital).

	Definite or probable diagnosis of COVID-19 vs. no COVID-19	
	**Model 1 [a] (N=137/644)**	**Model 2 [a] (N= 97/478)**
**Characteristics**	**OR [95%CI]**	**OR [95%CI]**
**Recruitment period**		
May 18–November 5, 2020	4.2 [1.4–12.6]	4.4 [1.2–15.5]
November 6, 2020–February 28, 2021	3.1 [1.0–9.2]	2.9 [0.8–10.3]
March 1, 2021–July 18, 2021	Ref.	Ref.
**Regional COVID-19 incidence per 100,000 inhabitants**		
<50	Ref.	Ref.
≥50	8.3 [3.8–17.9]	7.7 [3.2–18.7]
**COVID-19 contact in the community**		
No	Ref.	Ref.
Yes	3.0 [1.8–5.1]	2.4 [1.2–4.9]
**Workplace area**		
Emergency departments	2.2 [0.8–5.9]	1.5 [0.3–7.9]
Geriatric wards	4.2 [1.5–11.7]	3.9 [1.4–11.5]
Other specialty wards	3.0 [1.3–7.0]	2.7 [1.2–6.3]
Surgical wards	2.7 [0.9–7.4]	3.1 [0.9–8.7]
Intensive care units	Ref.	Ref.
**Working in a sector**		
With no COVID-19 patients	Ref.	-
With some COVID-19 patients	1.3 [0.8–2.1]	-
Dedicated to COVID-19 patients	1.8 [1.05–2.9]	
**Proportion of COVID-19 patients [b]**	-	1.8 [1.3–2.6]

Abbreviations: OR, odds ratio; CI, confidence interval. [a] OR for the association between the specified factor and COVID-19 using mixed-effects logistic regression (with a random intercept) after controlling for all other factors listed in the table and accounting for correlation within each hospital. [b] The proportion of COVID-19 patients was determined for all inpatient wards other than psychiatry, rehabilitation, and long-term care (N=579); ORs [95%CI] are quoted for one standard deviation increment in the log-transformed value.
